# Advancing beam shaping from visible light to X-rays for synchrotron applications

**DOI:** 10.1038/s41598-026-55263-9

**Published:** 2026-05-28

**Authors:** Hossein Khosroabadi, Pierpaolo Romano, Lucia Alianelli, Jose Brandao-Neto, Ralf Flaig, Kawal Sawhney

**Affiliations:** 1 Diamond Light Source, Harwell Science and Innovation Campus, Oxford, OX11 0DE UK; 2 Rutherford Appleton Laboratory, Research Complex at Harwell, Harwell, OX11 0FA UK

**Keywords:** beam shaping, top-hat profile, undulator tuning, refractive focusing optics, Optics and photonics, Physics

## Abstract

Customized beam shaping has a wide range of applications from visible light to hard X-rays. While laser beam shaping has matured over recent decades, enabling breakthroughs in optical communication, optical tweezers, and advanced microscopy, extending these techniques to high-brightness X-ray sources could significantly enhance synchrotron applications such as macromolecular crystallography, spectroscopy, and high-resolution imaging. However, X-ray beam shaping remains challenging due to limitations in the available optics and the finite phase-space of synchrotron sources. We introduce a novel method that exploits the monochromatic angular spectrum of undulator radiation combined with the compound refractive lenses (CRLs) to produce a variable circular focal spot with a top-hat intensity profile. By fine-tuning the undulator gap and monochromator settings, this approach enables dynamic control of the spatial beam profile while preserving continuous energy tunability within the limits imposed by the optical configuration and experimental conditions. This technique delivers flexible beam shaping without requiring complex new optical designs, construction, or operational overhead. This method has been successfully demonstrated on a macromolecular crystallography beamline at the Diamond Light Source (DLS), confirming its practicality, adaptability, and potential for widespread adoption in synchrotron-based research.

## Introduction

Laser beam shaping has evolved into a transformative tool for modern photonics, enabling precise control over spatial intensity, phase, and wavefront characteristics^1–7^. Recent conversion developments of conventional Gaussian beams into profiles such as flat-top^8^, Bessel^9^, ring-shaped^10^, and vortex modes^11^ have enhanced laser applications. Flat-top and Bessel beams are essential for various laser applications such as laser micro structuring^8^ and micromachining^12^, additive manufacturing^13^, precision cutting^14^ and microscopy ensuring optimized energy deposition during material processing. Vortex beams, which carry orbital angular momentum, have advanced optical communication^15^ and quantum information sciences^16^. The tailored profile has resulted in revolutionary applications such as optical communication^15^, optical tweezers with sub-micron precision^10^, high-resolution lithography, and biomedical imaging techniques including confocal and multiphoton microscopy. Currently there is considerable ongoing research to advance these techniques^17–19^.

Customized beam shaping in the X-ray regime is equally desirable for exploring matter at atomic and molecular scales. It is critical for the scientific output of synchrotron and free-electron laser facilities in techniques such as macromolecular crystallography (MX)^20–22^, imaging, and spectroscopy^23^. Structured X-ray illumination enhances coherent diffraction imaging, X-ray holography, and time-resolved pump–probe methods for nanoscale investigations of materials and biological specimens^24,25^. While matching the focal spot to the specimen size (e.g. crystals of biological molecules) significantly reduces background scattering and improves signal-to-noise ratio^20^. Uniform top-hat beam profiles, rather than Gaussian, minimize radiation damage to biomolecular crystals, extending crystal lifetime and improving structural determination accuracy^26,27^.

Visible-light beam shaping relies on mature methods such as refractive aspheric lenses^1^, diffractive optics (e.g., computer-generated holograms^28^, adaptive deformable mirrors^29^ and spatial light modulators (SLMs)^3^ capable of dynamic phase modulation. None of these techniques, however, can be straightforwardly applied at X-ray light source facilities. Fundamental differences, such as the sub-nm wavelength and strong energy-dependence of the complex refractive index in the X-ray range, $\:n=1-\delta\:+i\beta\:$^30^, make X-ray optics development inherently difficult. Although $\:\delta\:$ <<1 and $\:\beta\:\:$<<1, $\:\delta\:/\beta\:$ is small and therefore slight refraction is accompanied by high absorption. Thick phase shift plates, common in visible optics, are thus ineffective for X-rays. SLMs become unusable due to high absorption and nanometer-scale feature requirements. Grazing incidence reflection makes the reflected beam quality highly sensitive to tiny surface imperfections and impurities^31^. Diffractive optics such as zone plates^32^, kinoform lens^33,34^ or multilayer Laue lens^35^ suffer also from size constraints, low efficiency, and extreme sensitivity to the nanometer-scale fabrication errors. Practical issues like thermal loads, atomic impurities, and alignment precision further complicate X-ray optics implementation. X-ray Alvarez lens^36^ and achromatic lens^37^ development, decades after their visible-light counterparts, underscores the difficulty of extending visible-light innovations to X-ray systems.

Other fundamental constraints could originate from the intrinsic properties of light source facilities and their photon sources. Unlike lasers, X-ray beams generated from electron bunches have large phase-space and partial spatial coherence^38,39^ which limits interference-based beam shaping techniques. Furthermore, insertion devices introduce additional complication due to their relatively broad spectral bandwidth (ΔE/E > 10⁻^2^) and significant angular spread for the refractive or diffractive optics. The extended source size and mixed coherence state prevent formation of sharply structured profiles achievable with lasers.

Despite the limitations, innovations of kinoform^33,34^ and customized refractive^40^ lenses, multilayer Laue lens^35^, adaptively bent^41,42^ or customized surface profile mirrors^43–45^ have enabled huge scientific achievements through partial beam-shaping. Nanofocusing, improving coherence, real-time wavefront corrections^46^, and generation of top-hat profiles^43,44^ have supported critical experiments such as high-resolution coherent diffraction imaging^47^, ptychographic scanning microscopy^48^, advanced spectroscopies, and nanostructure characterization^49^. However, these optics are still suffering from unwanted beam structure^43,44^, high absorption, and micromachining challenges. They also often require design, fabrication, commissioning of the custom optics, and optimization routines which introduces additional technical and operational complexity to the densely populated synchrotron beamlines.

We propose a complementary novel approach that leverages the intrinsic angular spectrum of monochromatic undulator radiation^50–52^. Undulator sources are widely used in advanced light sources to maximize the photon brightness^51,52^ at their resonance frequencies, $\:{E}_{n}=n{E}_{1}$ where $\:{E}_{1}$ is the first resonance and n is an integer. While undulator radiation properties have long been known^53^, they have been used only for limited applications such as undulator commissioning^50,54,55^ and zone plate illumination^56^. Here, we expand the intrinsic undulator radiation features as an extra component for x-ray beam shaping at synchrotron facilities without requiring new optical components or complex feedback systems.

## Results and discussion

The concept of undulator gap tuning has emerged from the optical design studies for the new flagship Diamond-II (D-II) beamline K04^57^. This high-throughput MX beamline will require a variable size circular beam with a top-hat intensity profile. Achieving the requirements is a challenge with conventional optics such as bimorph Kirkpatrick-Baez (KB) mirrors or separate sets of 2D and 1D CRL lenses (see details in the Method section). To address the issues, a novel approach of undulator detuning is proposed.

**Fig. 1:**
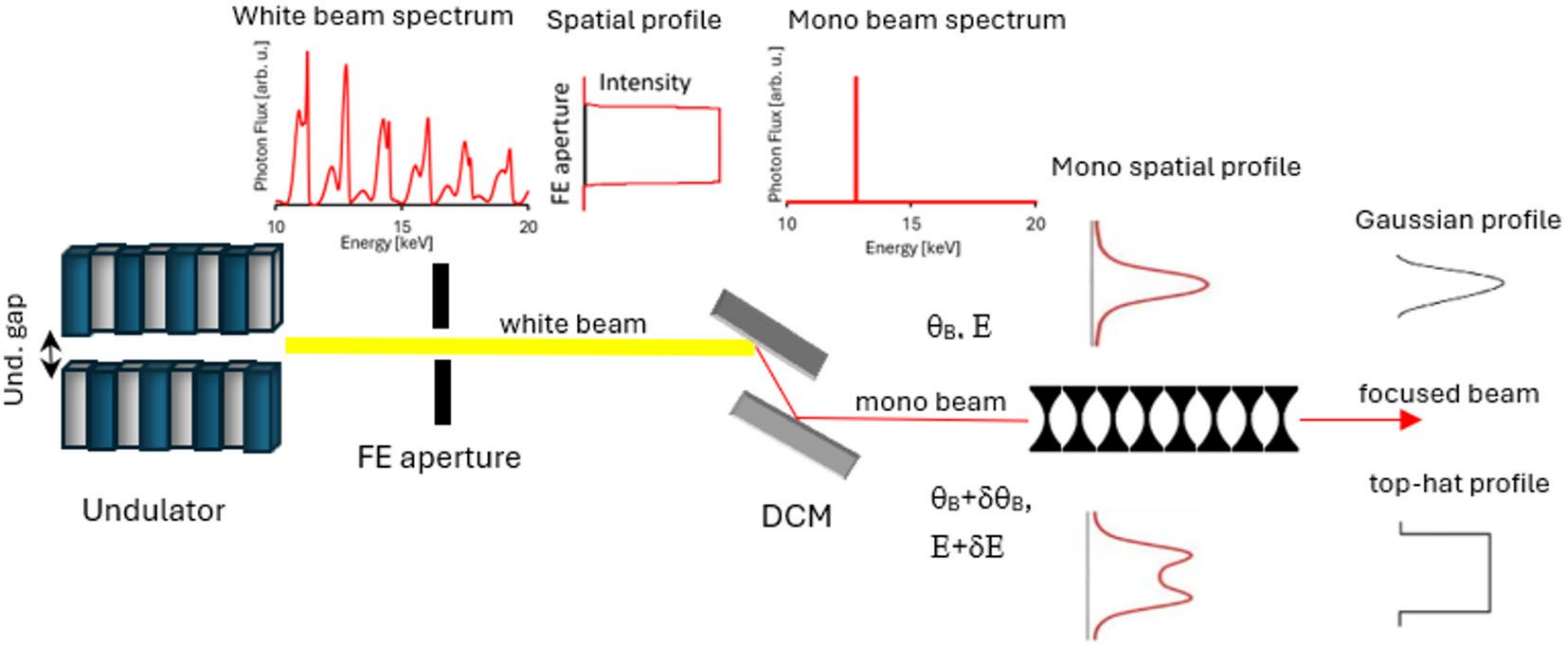
Schematic beamline layout consisting of undulator source, DCM monochromator, and CRL lenses. The resulting energy spectrum and spatial distribution are shown after the FE aperture (**a**), and after the DCM at two different Bragg angle (energy) settings. $\:{\theta\:}_{B}$ is the Bragg angle and *\:E* is the photon energy (**b**). Focus profile for two DCM energy settings in which a single peak or a split peak downstream of the DCM are transformed into a sharper Gaussian or a flat beam downstream of the CRL by tuning the splitting (**c**). The same beam profiles can be achieved by keeping the DCM energy setting constant while tuning the undulator gap.

Figure 1 illustrates the common schematic layout of two MX beamlines at DLS, i.e. K04 and I04^58^. Both use undulator radiation monochromatized by a double crystal monochromator (DCM) and focusing using CRL optics. CRL focusing optics are increasingly favoured in upgraded low-emittance light sources due to less striated beam at defocus positions^64^ compared to the reflective optics^43^. This represents a commonly employed layout at other synchrotron facilities for a variety of techniques such as MX^59,60^, nuclear resonance scattering^61^, high pressure crystallography^62^, and high energy diffraction^63^. The approach can be extended to these beamlines provided that several basic conditions are satisfied. It is applicable to hard X-ray beamlines based on in-vacuum linear undulators with a tuneable gap. More complex undulators, such as APPLE-II types commonly used for soft X-rays, are less suitable due to their complex magnetic field and polarization effects. Additional requirements include operation on low-vertical emittance storage rings that preserve the vertical double-peak structure, and utilizing low-absorption CRLs (e.g., Be or diamond) with apertures matched to the angular separation. F-switch CRLs^58^ can further facilitate energy detuning cases. Ray-tracing simulations are a powerful tool to provide a straightforward advice to assess compatibility with these criteria.

The relativistic electrons that pass through the periodic magnetic field of an undulator emit highly collimated beam cone of radiation at resonance energies of $\:{E}_{n}=n{E}_{1}$ which depend on both the undulator design and observation angle^53^. In practice, both the energy width and angular spread are broadened by machine parameters, electron beam emittance and undulator imperfections. The incident undulator radiation to the beamline is uniformly distributed at the front-end (FE) aperture (Fig. 1a). A typical DCM selects a narrow bandwidth (ΔE/E ~ 10^− 4^) of the incident white beam spectrum. The angular spectrum of the monochromatic beam is tuneable by slight detuning of the undulator gap or DCM setting. This offers a wide range of potential applications for beam shaping at the beamline end station. The angular spectrum can be analytically expressed by $\:{sin}^{2}\left(x\right)/{x}^{2}$^54,65^, similar to the well-known single-slit diffraction pattern, while the parameters are replaced by

1 $$\:x=N\pi\:(E-{E}_{n})/{E}_{1}$$

2 $$\:{E}_{1}\left(K,\theta\:,\psi\:\right)=A{\gamma\:}^{2}/{\lambda\:}_{u}(1+\frac{{K}^{2}}{2}+{\gamma\:}^{2}\left({\theta\:}^{2}+{\psi\:}^{2}\right))$$

*\:E*, *\:N*, *\:K*, $\:{\lambda\:}_{u}$, $\:\gamma\:$, and *\:A* are the DCM monochromatic energy, number of magnetic periods, undulator deflection parameter, magnetic period length, Lorentz factor, and a constant value, respectively. The angles $\:\theta\:$ and $\:\psi\:$ represent vertical and horizontal observation angles, respectively.

**Fig. 2:**
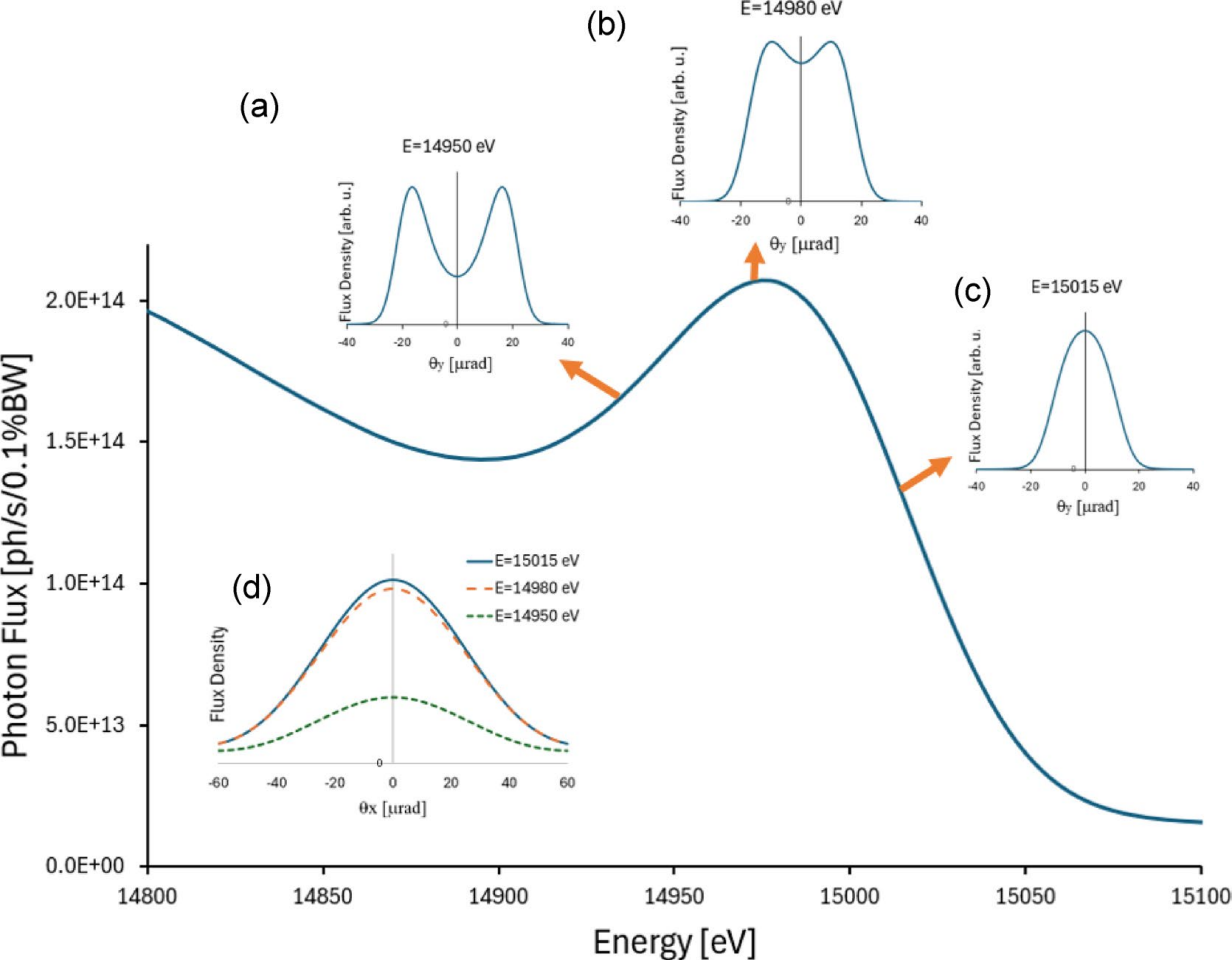
Spectrum of the 9th harmonic of a CPMU undulator in DLS machine downstream of the FE aperture calculated by SPECTRA^67^. The inset figures: (**a, b,** and** c**): vertical source profile at, respectively, the off-peak, peak flux and peak brightness at energies indicated by arrows, (**d**): horizontal source profile for the corresponding energies.

**Fig. 3:**
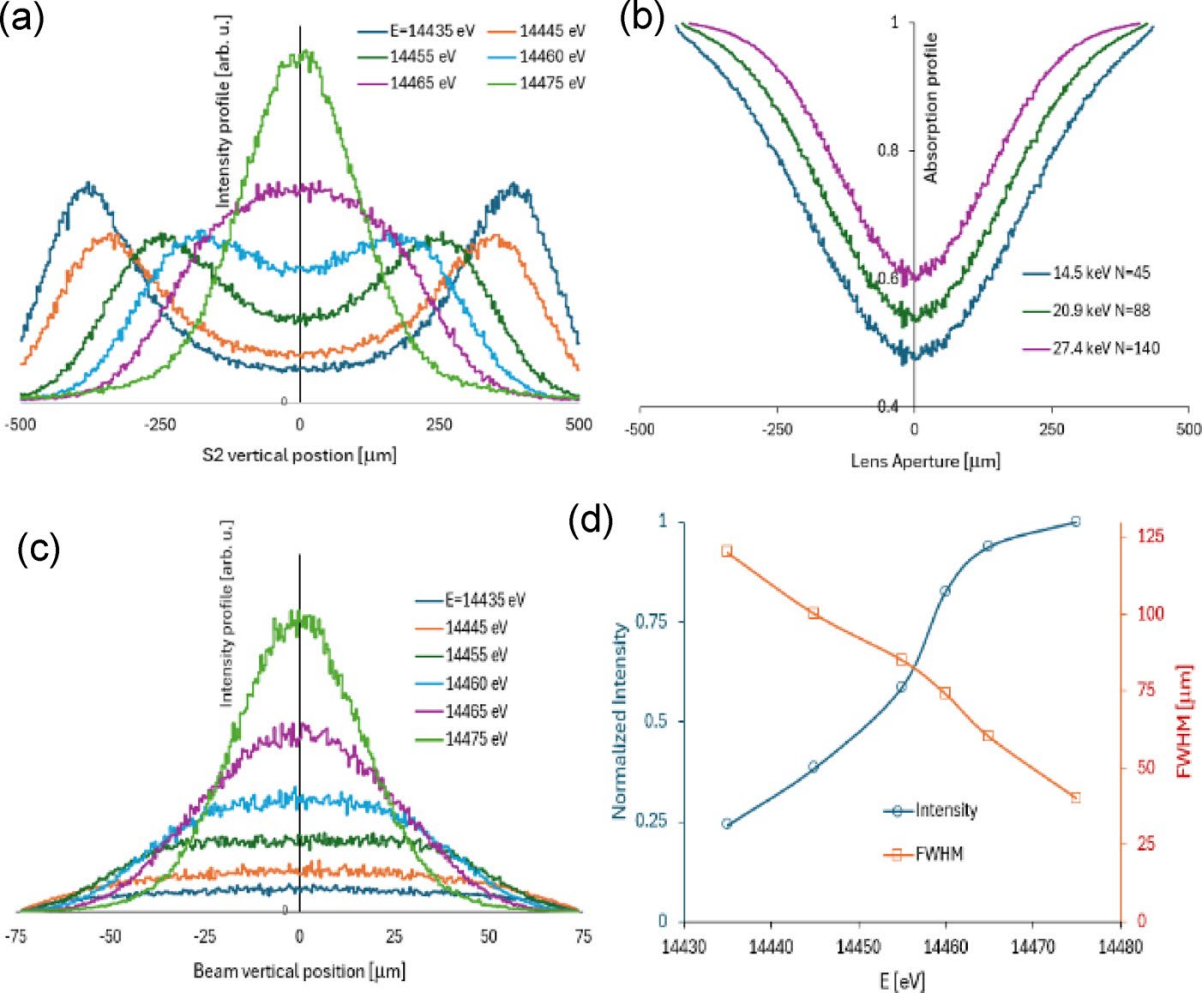
(**a**) CPMU undulator radiation profile in the vertical direction for tweaked energies calculated for a full undulator source by Shadow^52,68^. (**b**) Absorption profile for Be lenses at 14.5–27.4 keV energy range with different number of lenses (N) that yield a circular beam profile of 60 μm at the sample position. (**c**) Vertical beam profile at sample position for *N* = 45 lenses (900 μm physical aperture, 200 μm radius of curvature, 10 μm minimum thickness) in the corresponding detuned energy shown in (**a**). (**d**) FWHM beam size and normalized intensity inside the FWHM beam size vs. energy.

The Gaussian angular profile at the undulator resonances (peak brightness) evolves into a symmetric double-peak structure by shifting the energy downward as illustrated in Fig. 2. The angular profile is then converted into the spatial beam profile by the beamline’s focusing optics. Ideal reflective focusing optics normally preserve the incident shape, however refractive optics can reshape the beam due to their absorption profile. A top-hat profile can be achieved by matching the double-peak angular distribution to the absorption profile of the commonly used parabolic CRLs. The conversion is schematically shown in Fig. 1 and elaborated in Fig. 3. It should be noted that precise energy control (few eV) is essential to optimize the beam shaping in this method; therefore, wider band-width monochromators such as double-multilayer monochromators are not suitable for this method.

**Fig. 4:**
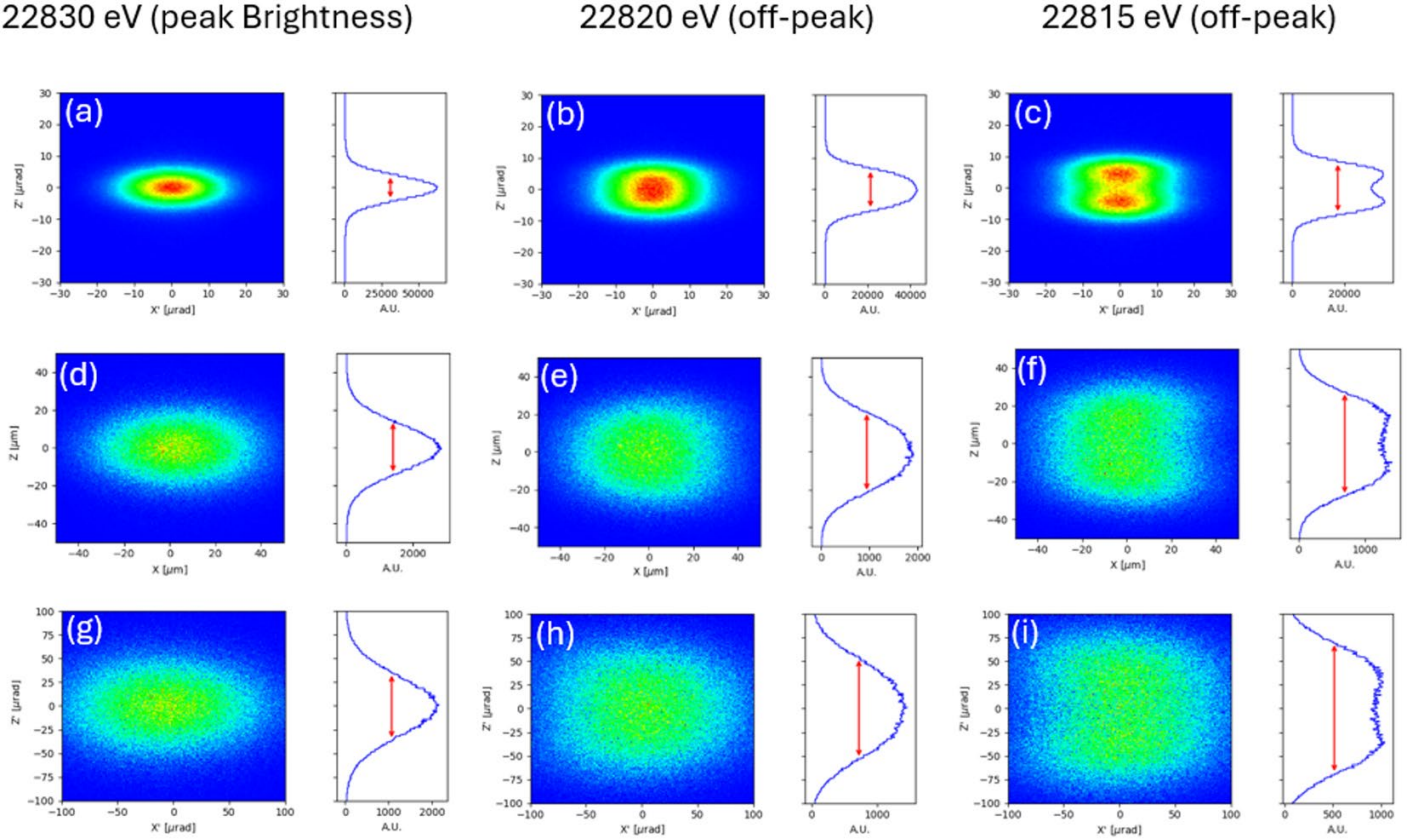
Prediction for achieving a rounded beam at the D-II K04 beamline at its 15th HPMU harmonic. Top row: Vertical source divergence of the HPMU undulator at different energies correspond to the peak brightness (**a**) and off peak (**b, c**) calculated by Shadow full undulator widget. Corresponding vertical beam size (**d-f**) and vertical beam divergence at sample (**g-i**) are shown. A rounded beam has been demonstrated in (**f**).

The vertical beam divergence is also tuneable, thereby reducing the beam divergence anisotropy and enabling variable and rounded beam at the end station. This method enables active beam shaping without the need for additional custom-designed optics and offers a practical solution for achieving circular beam and top-hat profile for I04 and K04 beamlines. Simultaneous undulator gap and DCM adjustment can provide an energy tuneable beam shaping, a useful concept for spectroscopy measurements. This offers a significant advantage over laser beam shaping, where the energy of the sources is not easily tuneable. Figure 2 simulates the angular evolution of the monochromatic beam from the CPMU at fixed gap. The double-peak structure emerges in the vertical direction near the peak flux (Fig. 2b) and separates by ~ ± 15 µrad at 50 eV below the resonance energy (Fig. 2a). So, beam divergence continuously changes to twice the divergence of a Gaussian profile, beneficial for rounded beam shaping (see Fig. 4) or focal size broadening as a practical alternative to the X-ray Alvarez lens recently proposed in Ref^36^. This also results in a larger optics illumination with advantages to the full-field transmission X-ray microscopy as proposed in Ref^56^. One can also notice that the monochromatic flux remains smooth at this energy range.

The angular separation between the double peaks increases continuously to reach ~ 3 times the undulator radiation cone half-width (Eq. 3) at a detuned energy of $\:{E}_{n}\left(1-1/Nn\right)$,

3 $$\:{\sigma\:}_{r{\prime\:}}\cong\:\sqrt{(1+\frac{{K}^{2}}{2})/2Nn{\gamma\:}^{2}}$$

For a given undulator and harmonic number, the angular separation is nearly independent of photon energy, enabling energy scans without recalibration of the DCM at each undulator gap. This can be confirmed by SPECTRA code^67^ simulations for a given undulator. In practice a look-up table can be made to tune the DCM angle for different undulator harmonics. A small modification in the number of lenses is required if the focal position is to be maintained at a fixed position. For example, a change of ± 1 lenses are required for a ± 500 eV energy scan at 15 keV, whereas 20 lenses are used for focusing. The F-switch system enables such energy scans by adding or removing a single lens within approximately 1 s.

For typical parameters of a 3 GeV machine, K = 2, *N* = 100, *n* = 7, this yields a peak separation range of ~ 23 µrad. This range is well matched to the available undulators and CRL aperture and its typical position in synchrotron beamlines. Commercial CRLs (beryllium, diamond or aluminium) have typical physical aperture ranges of ~ 450–900 μm. Their effective apertures are reduced to ~ 300–700 μm by the scaling factor of $\:\sqrt{\lambda\:f\delta\:/\beta\:}$^66^ where $\:\delta\:$ and $\:\beta\:$ are the imaginary and real part of the complex refractive index defined in Introduction, and $\:\lambda\:$ and *\:f* are the X-ray wavelength and focal length, respectively. In our case, given a lens distance of ~ 30 m from the source, the range corresponds to angular peak separations of 10–23 µrad.

Be, diamond, and Al materials mostly absorb X-rays below 5 keV, but the beam shaping concept remains valid at higher energies, 10–30 keV, due to the higher number of lenses required (see Fig. 3b). Optimal matching can be found by fine tuning the DCM Bragg angle for a set of lenses as shown in Fig. 3c. The long tail can then be trimmed using a downstream slit without considerable loss of photon flux at sample. Figure 3d presents the expected flux at the top-hat profile, which is approximately half of the flux at peak brightness.

Figure 4 presents ray-tracing analysis for the K04 beamline, revealing an elliptical focal spot of 6 μm x 1 μm (HxV, FWHM). At the defocused position, the beam remains anisotropic, i.e. 49 μm x 28 μm as shown in Fig. 4d. By tuning the undulator angular spectrum, the beam converts into a rounded shape (50 μm x 55 μm) with isotropic beam divergence (Fig. 4i and f). Controlling the beam spot by its divergence provides interesting consequences such as achieving rounded beam and top-hat shape for variable distance from the focal position without disturbing optics. This is confirmed across the whole energy range (10–27 keV) for the K04 beamline.

**Fig. 5:**
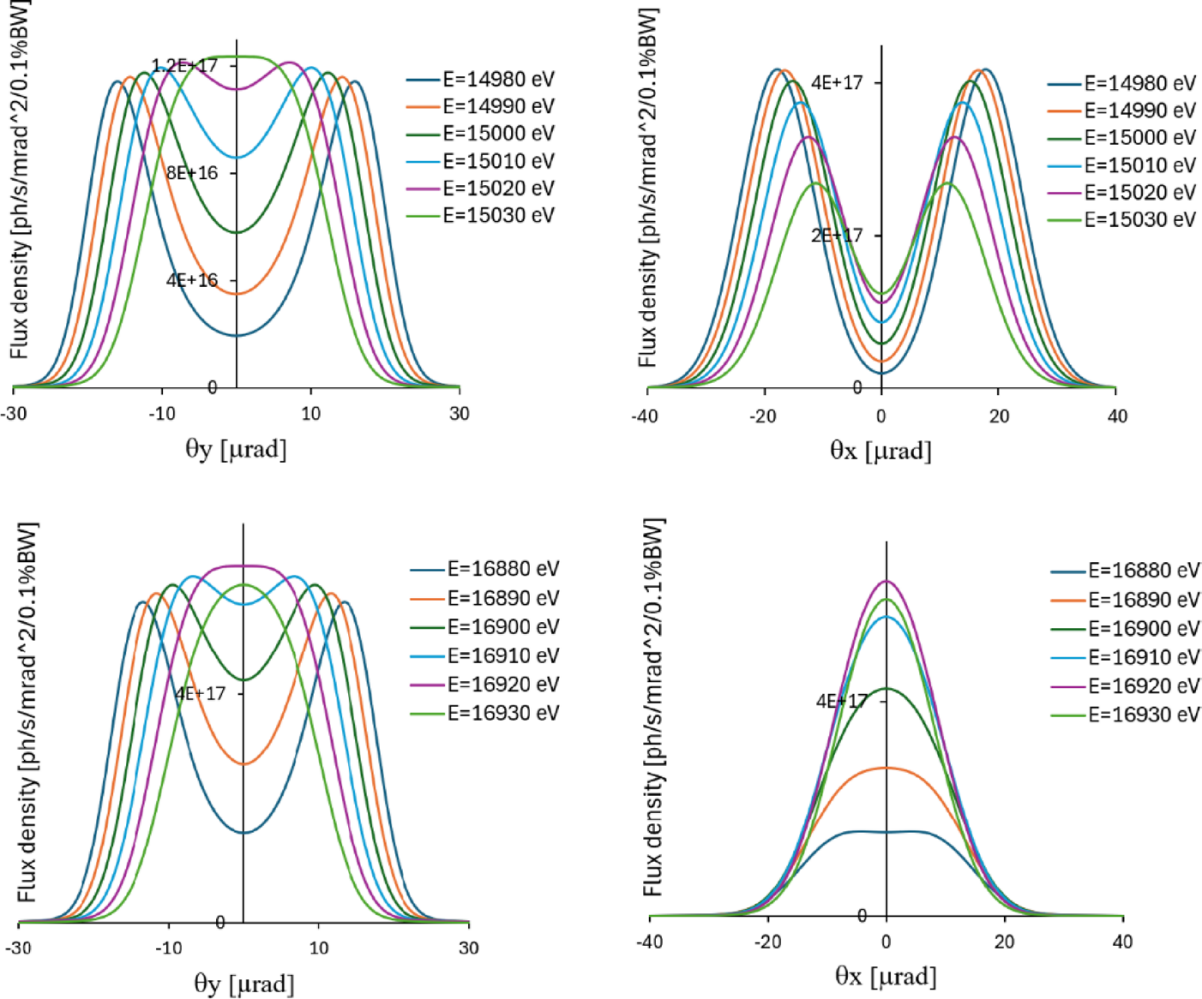
Angular spectrum for the I04 CPMU source in D-II machine for 8th (top) and 9th (bottom) harmonics calculated by SPECTRA^67^. Plots against horizontal angle are on the right, and plots against vertical angle are on the left. The double-peak structure in the horizontal direction happens at the 8th harmonic but not the 9th .

Extending the concept to the horizontal direction to obtain a 2D top-hat profile requires further investigation. Peak splitting in a linear undulator depends on the number (even vs. odd) of the undulator harmonic, observation direction (horizontal vs. vertical), and electron beam emittance. Odd harmonics show splitting in the vertical direction, while even harmonics can show it in both directions. A large electron beam emittance can blur the split peaks into a broadened single peak. This is the case for the large horizontal emittance of the current DLS machine and the upgraded D-II machine^57^. SPECTRA simulations shown in Fig. 5 confirm the bidirectional splitting for the D-II machine. To achieve a balanced 2D top-hat profile an optimal vertical and horizontal angular spectrum should be identified. Two alternative solutions are proposed for machines with large horizontal emittance: (1) horizontal defocusing by replacing some of the 2D lenses with 1D vertical lenses, (2) hybrid focusing using 1D vertical lenses and a horizontal focusing elliptical mirror^59^ to convert the flat part of the horizontal angular spectrum. Ray tracing analysis confirms both solutions.

**Fig. 6:**
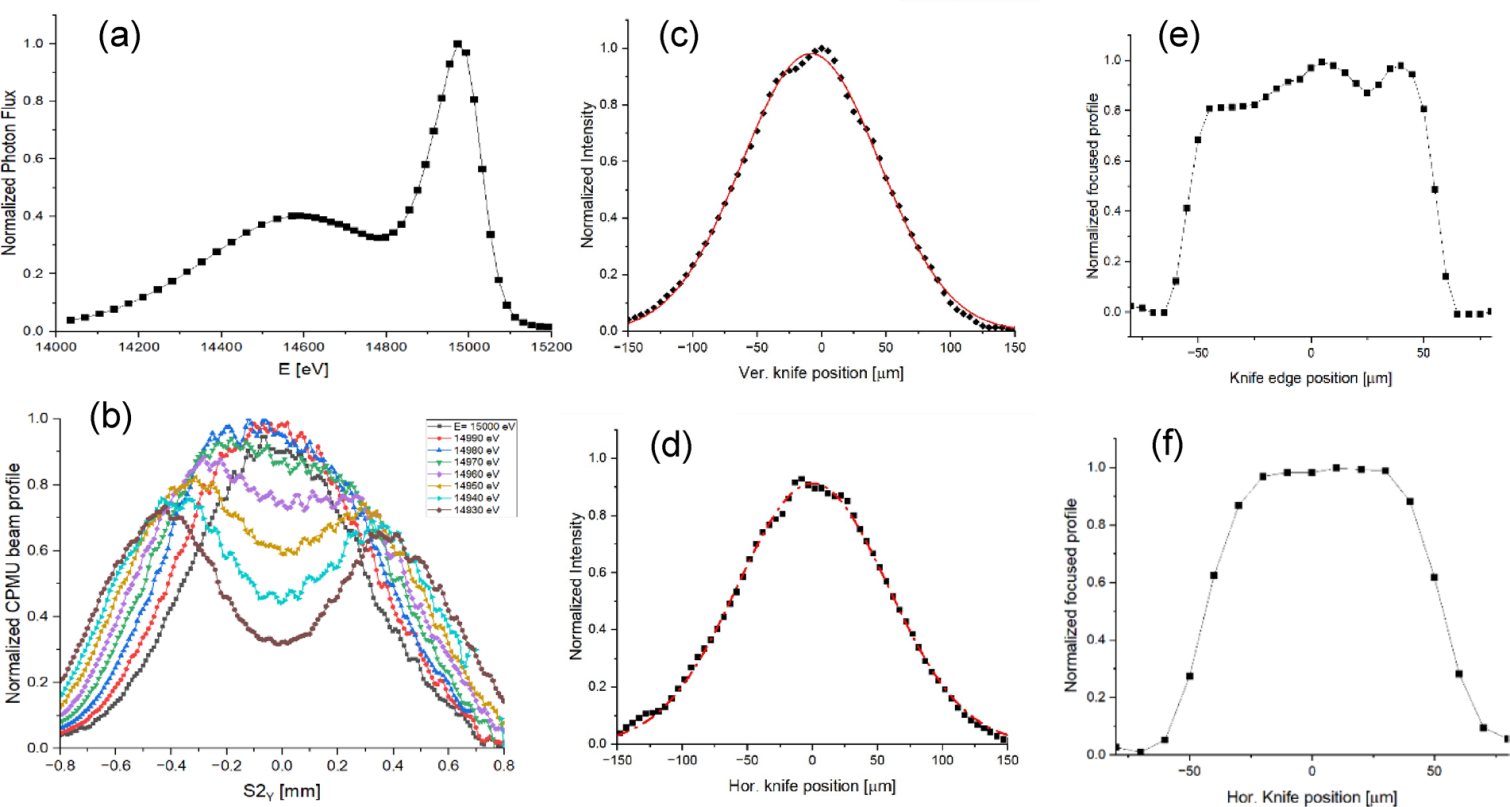
(**a**) Normalized measured CPMU flux, and (**b**) beam profile in the vertical direction at slit S2 of beamline I04 for a range of energies around peak flux (14980 eV). Knife edge scans at I04 sample position: (**c**) vertical and (**d**) horizontal Gaussian beam profile at the peak brightness, and (**e**) vertical and (**f**) horizontal top-hat beam profile measured in the reduced lens configuration. The dots represent the measuerd data, the solid red lines indicate Gussian fits to the data, and dashed lines are guide to the eye.

To validate the elaborated beam shaping concept, a series of measurements were conducted on beamline I04. I04 is equipped with CPMU source, DCM monochromator, and CRL focusing optics. The CRLs are housed in a F-switch device that allows individual lens control in and out of the beam path to achieve beam sizes from sub-micron to 100 μm in the vertical dimension^58^. Two active diagnostic feedback systems dynamically align the DCM and F-switch optics with the undulator beam. Figure 6b illustrates the continuous evolution of double-peak structure of the monochromatic beam at the downstream S2 slit position. The required double-peak splitting for top-hat occurs at ~ 50 eV off peak. The profiles appear slightly asymmetric, which may originate from a combination of tiny misalignments (in vertical positioning or incidence angle) of the electron beam relative to the undulator axis^50^. Knife-edge scans at the sample position, revealed a gradual transition from a Gaussian peak to a top-hat profile at a specific energy. Both the Gaussian beam at the peak brightness and top-hat profile in the vertical and horizontal directions are shown in Fig. 6c and f. Both vertical and horizontal top-hat profiles were achieved in a reduced-lens configuration, obtained by removing few lenses from the beam path on the DLS large-emittance machine. A slight adjustment of the energy or undulator gap is required to have the vertical top-hat in the reduced lens configuration.

The resulting top-hat profiles are smoother than those produced using static surface profiling^44^ or bimorph mirror shaping^41,43^ . The vertical top-hat profile exhibits a slight tilt, likely arising from asymmetries in the spatial distribution of the undulator radiation, and increased noise associated with the strong CRL focusing used in this demonstration. We believe the observed asymmetry arises from instrumental factors (electron beam orbit and strong CRL focusing) rather than from a fundamental limitation of the method. These effects can be mitigated by optimizing the undulator alignment to the beam orbit and by reducing the number of lenses in layouts with longer focal distances. Misalignment between the undulator axis and the electron beam, affecting both white-beam and monochromatic radiation, has been widely studied in the literature^50,70,71^. These help to establish alignment strategies to mitigate the asymmetries in the undulator radiation and, consequently, in the top-hat profile. An asymmetry at the ~ 10% level is acceptable for typical macromolecular crystallography applications; however, experiments requiring higher spatial uniformity may necessitate additional correction.

The measured flux at the sample in peak-flux and top-hat condition is 9 × 10^11^ ph/s and 2 × 10^11^ ph/s, respectively, at 15 keV. Although the reduced flux remains still adequate for typical biological crystallography experiments, considering the current detector speed limitations and finite crystal lifetime, it may pose a constraint for photon-hungry techniques. The flux can be substantially recovered by increasing the CRL acceptance and reducing the number of lenses in use. It should be emphasized that the present strong focusing CRL in I04 serves primarily as a proof-of-concept for top-hat formation. Future developments at I04 beamline aim to offer 2D top-hat beams in a user-friendly mode, thereby removing the flux reduction limitations. To our knowledge, comparable flux values have not been systematically reported for alternative top-hat methods. Ideal top-hat profile generated using a KB bimorph system is expected to deliver flux levels similar to those of a Gaussian beam. In practice, however, mitigation of pronounced beam striations often requires mirror aperture reduction, leading to a considerable loss of flux. Moreover, residual profile imperfections further limit the attractiveness of the bimorph approach. In comparison, CRL-based optics offer a more flexible and robust route to achieving reliable top-hat profiles, whereas KB bimorph shaping is subject to substantial technical challenges, including complex mirror deformation control and the requirement for costly active feedback system^41^. Therefore, the choice of top-hat beam strategy should carefully balance the trade-off between achieved high spatial uniformity or maintaining high flux for the intended beamline applications.

In summary, we have developed and validated a novel method for X-ray beam shaping using the intrinsic angular spectrum of undulator radiation, a crystal monochromator, and refractive focusing optics. This approach enables tuneable focus size, rounded beam shape, and top-hat profile, which are ideal for macromolecular crystallography. Crucially, it requires adjustment of only two parameters: the undulator gap and monochromator energy setting, without the need for new optics. In principle, the method can be generalized to other beamlines with a similar layout, provided its merits and technical limitations are carefully evaluated. The choice of top-hat beam strategy should carefully balance the trade-off between achieved high spatial uniformity or maintaining high flux. Overall, this method enhances beamline versatility and opens new opportunities for high-precision X-ray applications.

### Method

This beam shaping concept was developed for two MX beamlines at Diamond Light Source, I04 and K04, and experimentally validated on I04.

**Fig. 7:**
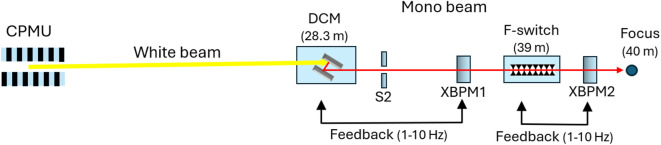
Schematic layout of I04 beamline at DLS^58^ used for top hat beam measurement.

I04 is an operational MX beamline equipped with a recently upgraded cryogenic permanent magnet undulator (CPMU) source with magnetic period of 17.6 mm. I04 layout is shown in Fig. 7. The X-ray beam is monochromatized using a Si(111) DCM and focused by a F-switch CRLs system located 39 m from the source containing 2D Be lenses. Two active diagnostic feedback loops are employed to dynamically align both the DCM and F-switch optics, ensuring precise beam delivery to the sample position at 40 m from the source^58^. To characterize the undulator monochromatic spatial beam profile, slit-edge scanning was performed at the S2 position 30 m downstream of the source. Knife-edge scans, using a gold blade of 25 μm thickness (manufactured by Arndt Last at Karlsruhe Institute of Technology (KIT)), at the sample position were conducted across various undulator configurations, harmonics, and both horizontal and vertical directions to measure beam size and shape. These scans covered undulator harmonics in the 7–18 keV energy range. A larger undulator gap was selected during extended scans to minimize thermal deformation effects on the DCM. While not strictly necessary, this precaution complements the use of diamond filters, which help mitigate heat load effects. During measurements, the feedback system was temporarily disabled to prevent unintended beam shifts relative to the CRL lenses.

K04, now under design, is an ultra-high throughput MX beamline, one of the flagship beamline projects forming part of the D-II upgrade, intended to replace the existing single wavelength I04-1 beamline. K04 will use a hybrid permanent magnet undulator (HPMU), currently in use as I04-1 source. In K04 the HPMU will be followed by a horizontal-bounce DCM and a F-Switch transfocator for beam focusing. The spectral and spatial photon distribution of the CPMU and HPMU sources were simulated using SPECTRA^67^ and full-undulator widget in Shadow under OASYS platform ^68^. Ray-tracing simulation for various undulator detuning scenarios were conducted using Shadow^52,68,69^ to evaluate beam shaping performance. K04 presents a highly anisotropic source profile, with a source size of 64 × 10 µm^2^ and divergence of 20 × 8 µrad^2^ (FWHM, HxV). Multiple optical configurations were assessed to meet the beamline’s requirement for a circular beam with variable size ranging from 5 to 50 μm. Simulations predict a large horizontal divergence (~ 1 mrad) and strong induced-beam structure in defocused condition for a bendable KB mirrors layout, which would result in degraded data quality. 2D Be lenses yielded acceptable beam divergence but resulted in an elliptical focal spot with 2-6x anisotropy. Separate sets of 2D and one-dimensional (1D) lenses, as an alternative, faces the practical concern of time-consuming alignment, which is incompatible with the beamline’s goal of ultra-high sample throughput. The undulator-DCM-CRL setup, on the other hand, yields round, flat X-ray beam profiles and uses only standard components that are simple to align.

## Data Availability

Data supporting the findings of this study are available from the manuscript or from the corresponding author upon request.

## References

[1] Zhang, S., Yang, Q. & Lüpke, G. Spatial beam shaping of ultrashort laser pulses: theory and experiment. *App Opt.* **44**, 5818 (2005). 10.1364/ao.44.005818 16201448

[2] Kim, J. H. & Choi, H. W. Review on Principal and Applications of Temporal and Spatial Beam Shaping for Ultrafast Pulsed Laser. *Photonics* **11**, 1140 (2024).

[3] Ngcobo, S., Litvin, I., Burger, L. & Forbes, A. A digital laser for on-demand laser modes. *Nat. Commun.* **4**, 2289 (2013). 23907358 10.1038/ncomms3289

[4] Wei, D. et al. Efficient nonlinear beam shaping in three-dimensional lithium niobate nonlinear photonic crystals. *Nat. Commun.* **10**, 4193 (2019). 31519901 10.1038/s41467-019-12251-0 PMC6744429

[5] Möhl, A. et al. Tailored focal beam shaping and its application in laser material processing. *J. Laser Appl.* **31**, 042019 (2019).

[6] Ji, W. et al. & Y.-Q. L, Meta-q-plate for complex beam shaping, Sci. Reports **6**, 25528 (2016). 10.1038/srep25528 PMC4858674 27149897

[7] Mauclair, C., Najih, B., Comte, V., Bourquard, F. & Delaigue, M. Dynamic spatial beam shaping for ultrafast laser processing: a review. *Opto-Electron Sci.* **4**, 250002 (2025).

[8] Le, H. et al. Effects of Top-hat Laser Beam Processing and Scanning Strategies in Laser Micro-Structuring. *Micromachines* **11**, 221 (2020). 32093369 10.3390/mi11020221 PMC7074761

[9] Duocastella, M. & Arnold, C. B. Bessel and annular beams for materials processing. *Laser Photonics Rev.* **6**, 607 (2012).

[10] Būtaitė, U. G. et al. Photon-efficient optical tweezers via wavefront shaping. *Sci. Adv.* **10**, 7792 (2024). 10.1126/sciadv.adi7792 PMC11225778 38968347

[11] Coursault, D. & Brasselet, E. Nanostructured silica spin–orbit optics for modal vortex beam shaping. *Nanophotonics* **11**, 805 (2022). 39635373 10.1515/nanoph-2021-0579 PMC11501947

[12] Baltrukonis, J., Ulcinas, O., Orlov, S. & Jukna, V. High-Order Vector Bessel-Gauss Beams for Laser Micromachining of Transparent Materials. *Phys. Rev. Appl.* **16**, 034001 (2021).

[13] Tumkur, T. U. et al. Nondiffractive beam shaping for enhanced optothermal control in metal additive manufacturing. *Sci. Adv.* **7**, 9358 (2021). 10.1126/sciadv.abg9358 PMC8443179 34524849

[14] Rodrigues, G. C. & Duflou, J. R. Into the development of a model to assess beam shaping and polarization control effects on laser cutting. *J. Phys. D: Appl. Phys.* **51**, 065601 (2018).

[15] Liverman, S. et al. Dynamic indoor free-space optical communication enabled by beam steering and beam shaping. *Appl. Opt.* **62**, 2367 (2023). 37132876 10.1364/AO.451422

[16] Leach, J. et al. Quantum Correlations in Optical Angle–Orbital Angular Momentum Variables. *Science* **329**, 662 (2010). 20689014 10.1126/science.1190523

[17] Zeng, Y. et al. Metalasers with arbitrarily shaped wavefront. *Nature* **643**, 1240 (2025). 40634607 10.1038/s41586-025-09275-6

[18] Aït-Ameur, K. The Advantages and Disadvantages of Using Structured High-Order but Single Laguerre–Gauss *LGp*_0_ Laser Beams. *Photonics* **11**, 217 (2024).

[19] Chen, Y., Wang, F. & Cai, Y. Partially coherent light beam shaping via complex spatial coherence structure engineering. *Adv. Phys. : X*. **7**, 2009742 (2022).

[20] Grimes, J. M. et al. Where is crystallography going? *Acta Cryst.* **D74**, 152 (2018). 10.1107/S2059798317016709 PMC5947779 29533241

[21] Zeldin, O. B., Gerstel, M. & Garman, E. F. Optimizing the spatial distribution of dose in X-ray macromolecular crystallography. *J. Syn Rad*. **20**, 49 (2013). 10.1107/S0909049512044706 23254655

[22] Paithankar, K. S., Owena, R. L. & Garman, E. F. Absorbed dose calculations for macromolecular crystals: improvements to RADDOSE. *J. Syn Rad*. **16**, 152 (2009). 10.1107/S0909049508040430 19240327

[23] Vogel-Mikuš, K. Synchrotron X-ray Fluorescence Imaging and Recent Developments, Chapter, 77–112 In: (ed Singh, V. K.) X-Ray Fluorescence Spectroscopy and Chemometrics. Springer (2025).

[24] Greenberg, J. A., Hassan, M., Krishnamurthy, K. & Brady, D. Structured illumination for tomographic X-ray diffraction imaging. *Analyst* **139**, 709 (2014). 24340351 10.1039/c3an01641b

[25] Sung, D. et al. Single-Shot Coherent X-ray Imaging Instrument at PAL-XFEL. *Appl. Sci.* **11**, 5082 (2021).

[26] Garman, E. F. Radiation damage in macromolecular crystallography: what is it and why should we care? *Acta Cryst.* **D66**, 339 (2010). 10.1107/S0907444910008656 PMC2852297 20382986

[27] Garman, E. F. & Weik, M. Radiation damage to biological macromolecules. *Curr. Opin. Struc Biology*. **82**, 102662 (2023). 10.1016/j.sbi.2023.102662 37573816

[28] Kuroo, N. & Hayasaki, Y. Design framework of a computer-generated hologram that performs volumetric beam shaping for advanced laser processing. *Opt. Continuum*. **3**, 1244 (2024).

[29] Bremer, S. J. L., Aarts, R. G. K. M. & Römer, G. R. B. E. Design and implementation of dynamic beam shaping in high power laser processing by means of a Deformable Mirror. *Opt. Laser Techno*. **177**, 111066 (2024).

[30] Attwood, D. *Soft X-rays and Extreme Ultraviolet Radiation: Principles and Applications* (Cambridge University Press, 1999).

[31] Salditt, T. & Osterhoff, M. in *X-ray Focusing and Optics, chapter in Nanoscale Photonic Imaging*. (eds Salditt, T.) A. Egner & D. Russell Luke,71–124 (2020).

[32] Wu, S. R., Hwu, Y. & Margaritondo, G. Hard–X–ray Zone Plates: Recent Progress. *Materials* **5**, 1752 (2012).

[33] Karvinen, P. et al. Kinoform diffractive lenses for efficient nano-focusing of hard X-rays. *Opt. Exp.* **22**, 16676 (2014). 10.1364/OE.22.016676 25090486

[34] Tiwari, M. K., Alianelli, L., Dolbnya, I. P. & Sawhney, K. Application of kinoform lens for X-ray reflectivity analysis. *J. Syn Rad*. **17**, 237 (2010). 10.1107/S0909049509055009 20157277

[35] Murray, K. T. et al. Multilayer Laue lenses at high X-ray energies: performance and applications. *Opt. Exp.* **27**, 7120 (2019). 10.1364/OE.27.007120 30876283

[36] Dhamgaye, V. et al. Alvarez varifocal X-ray lens. *Nat. Commun.* **14**, 4582 (2023). 37524749 10.1038/s41467-023-40347-1 PMC10390599

[37] Kubec, A. et al. An achromatic X-ray lens. *Nat. Commun.* **13**, 1305 (2022). 35288546 10.1038/s41467-022-28902-8 PMC8921332

[38] Nash, B., Goldring, N., Edelen, J. & Webb, S. Propagation of Partially Coherent Radiation Using Wigner Functions. *Phys. Rev. Accel Beams*. **24**, 010702 (2021).

[39] Vartanyants, I. A. & Singer, A. *Coherence Properties of Third-Generation Synchrotron Sources and Free-Electron Lasers, in Coherent X-Rays and Their Applications* (Springer, 2020).

[40] Markus, O. et al. Optimizing illumination for full field imaging at high brilliance hard X-ray synchrotron sources. *Opt. Exp.* **26**, 30435 (2018). 10.1364/OE.26.030435 30469917

[41] Alcock, S. G. et al. Fast shaping control of x ray beams using a closed-loop adaptive bimorph deformable mirror. *Optica* **10**, 172 (2023).

[42] Cocco, D. et al. Adaptive shape control of wavefront-preserving X-ray mirrors with active cooling and heating. *Opt. Exp.* **28**, 19242 (2020). 10.1364/OE.394310 32672205

[43] Sutter, J. P. et al. Creating flat-top X-ray beams by applying surface profiles of alternating curvature to deformable piezo bimorph mirrors. *J. Syn Rad*. **23**, 1333 (2016). 10.1107/S1600577516013308 PMC5082462 27787239

[44] Laundy, D., Alianelli, L., Sutter, J., Evans, G. & Sawhney, K. Surface profiling of X-ray mirrors for shaping focused beams. *Opt. Exp.* **23**, 1576 (2015). 10.1364/OE.23.001576 25835915

[45] Wang, H., Sutter, J. & Sawhney, K. Advanced in situ metrology for X-ray beam shaping with super precision. *Opt. Exp.* **23**, 1605 (2015). 10.1364/OE.23.001605 25835918

[46] Pan, X., Zuo, H., Bai, H., Wu, Z. & Cui, X. Real-time wavefront correction using diffractive optical networks. *Opt. Exp.* **31**, 1067 (2023). 10.1364/OE.478492 36785149

[47] Chushkin, Y. & Zontone, F. Prospects for coherent X-ray diffraction imaging at fourth-generation synchrotron sources. *IUCrJ* **12**, 280 (2025). 40080160 10.1107/S2052252525001526 PMC12044861

[48] Pfeiffer, F. X-ray ptychography. *Nat. Photonics*. **12**, 9 (2018).

[49] Berkowicz, S. et al. Nanofocused X-ray photon correlation spectroscopy. *Phys. Rev. Res.* **4**, L032012 (2022).

[50] Feng, C. et al. Coherent photon beam-based diagnostics for a seeded extreme ultraviolet free-electron laser. *Phys. Rev. Spec. Top. Acc. Beams*. **17**, 100702 (2014).

[51] Walker, R. P. Undulator radiation brightness and coherence near the diffraction limit. *Phys. Rev. Acc. Beams*. **22**, 050704 (2019).

[52] Sanchez del Rio, M. & Reyes-Herrera, J. Modelling undulators in ray tracing simulations. *J. Syn Rad*. **32**, 340 (2025). 10.1107/S1600577525000190 PMC11892896 39946282

[53] Clarke, J. A. *The Science and Technology of Undulators and Wigglers* (Oxford Series on Synchrotron Radiation, 2004).

[54] Ilinski, P. Undulator A Diagnostics at the Advanced Photon Source, Argon National Laboratory, ANL/APS/TB-33 report (1998).

[55] Eugene Tan, Y. R. et al. Martin Krichler, Design and commissioning of the first superconducting undulator for the BioSAXS beamline at the Australian Synchrotron. *J. Syn Rad*. **32**, 908 (2025). 10.1107/S1600577525003418 PMC12236254 40455638

[56] Wang, S. et al. Zone plate-based full-field transmission X-ray microscopy beamline design at nearly diffraction-limited synchrotron radiation facility. *Nuc Ins Met. Phs Res. A*. **993**, 165089 (2021).

[57] Chapon, L. C. et al. Diamond-II: Conceptual Design Report, Diamond Light Source, Oxfordshire, UK (2019).

[58] Romano, P., Khosroabadi, H., Aragao, D. & Flaig, R. Microfocus and variable beam size at the Diamond Light Source I04 beamline. *J. Phys. : Conf. Ser.* **3010**, 012051 (2025).

[59] McCarthy, A. A. et al. Mueller-Dieckmann, ID30B – a versatile beamline for macromolecular crystallography experiment at the ESRF. *J. Syn Rad*. **25**, 1249 (2018). 10.1107/S1600577518007166 PMC6038607 29979188

[60] Liermann, H. P. et al. The Extreme Conditions Beamline P02.2 and the Extreme Conditions Science Infrastructure at PETRA III. *J. Syn Rad*. **22**, 908 (2015). 10.1107/S1600577515005937 PMC4489534 26134794

[61] Rüffer, R. & Chumakov, A. I. Nuclear Resonance Beamline at ESRF. *Hyperfine Interact.* **97**, 589 (1996).

[62] Merlini, M. & Hanfland, M. Single-crystal diffraction at megabar conditions by synchrotron radiation. *High. Press. Res.* **33**, 511 (2013).

[63] Zimmermann, M. et al. P21.1 at PETRA III – a high-energy X-ray diffraction beamline for physics and chemistry. *J. Syn Rad*. **32**, 802 (2025). 10.1107/S1600577525002826 PMC12067326 40261645

[64] Celestre, R. et al. Modelling phase imperfections in compound refractive lenses. *J. Synch Rad*. **27**, 305 (2020). 10.1107/S1600577519017235 PMC7842213 32153269

[65] Howells, M. R. & Kincaid, B. M. in *The Properties of Undulator Radiation, Chapter in New Directions in Research with Third-Generation Soft X-ray Synchrotron Radiation Sources*. NATO ASI SeriesVol. 254, 315–358 (eds Schlachter, A. S. & Wuilleumier, F. J.) (Springer, 1994).

[66] Kohn, V. G. Effective aperture of X-ray compound refractive lenses. *J. Syn Rad*. **24**, 609 (2017). 10.1107/S1600577517005318 28452752

[67] Tanaka, T. Major upgrade of the synchrotron radiation calculation code SPECTRA. *J. Syn Rad*. **28**, 1267 (2021). 10.1107/S1600577521004100 34212893

[68] Sánchez del Río, M. & Rebuffi, L. OASYS: A software for beamline simulations and synchrotron virtual experiments. AIP Conf. Proc. 060081 (2019). (2054).

[69] Sanchez del Rio, M. New challenges in ray tracing simulations of X-ray optics. *J. Phys. : Conf. Ser.* **425**, 162003 (2013).

[70] Tanaka, T. et al. Undulator commissioning by characterization of radiation in x-ray free electron lasers. *Phys. Rev. Spec. Top. Accel Beams*. **15**, 110701 (2012).

[71] Chubar, O. et al. Spectrum-Based Alignment of In-Vacuum Undulators in a Low-Emittance Spectrum-Based Storage Ring. *Syn Rad News*. **31**, 4 (2018).

